# Diagnostic utility of the insulin-to-C-peptide molar ratio in differentiating insulin autoimmune syndrome and exogenous insulin antibody syndrome from insulinoma

**DOI:** 10.3389/fendo.2026.1879250

**Published:** 2026-07-07

**Authors:** Yang Wang, Yiwen Liu, Jie Yu, Baodi Xing, Fan Ping, Wei Li, Lingling Xu, Ming Li, Huabing Zhang, Yuxiu Li

**Affiliations:** Department of Endocrinology, Key Laboratory of Endocrinology of the National Health Commission, Peking Union Medical College Hospital, Chinese Academy of Medical Sciences and Peking Union Medical College, Beijing, China

**Keywords:** autoimmune hypoglycemia, diagnostic cutoff, exogenous insulin antibody syndrome, insulin autoimmune syndrome, insulinoma, insulin-to-C-peptide molar ratio

## Abstract

**Objective:**

Although an insulin-to-C-peptide molar ratio greater than 1 has traditionally been considered suggestive of insulin autoimmune syndrome (IAS), its diagnostic value remains controversial, and the optimal cutoff is unclear. This study aimed to evaluate the diagnostic performance of the insulin-to-C-peptide molar ratio for distinguishing IAS and exogenous insulin antibody syndrome (EIAS) from insulinoma and to identify the optimal diagnostic cutoff.

**Methods:**

We retrospectively collected clinical and biochemical data from 19 patients with IAS, 33 with EIAS, and 70 control patients with pathologically confirmed insulinoma. Receiver-operating-characteristic (ROC) curve analysis was performed separately for IAS versus insulinoma and EIAS versus insulinoma to assess the diagnostic performance of the insulin-to-C-peptide molar ratio in the fasting state and during hypoglycemic episodes.

**Results:**

The insulin-to-C-peptide molar ratio was significantly higher in IAS and EIAS than in insulinoma at both fasting and during hypoglycemic episodes. During hypoglycemic episodes, the area under the ROC curve (AUC) was 0.970 for IAS versus insulinoma and 0.944 for EIAS versus insulinoma, with optimal cutoffs of 0.382 and 0.552, respectively. In the fasting state, the corresponding AUCs were 0.916 and 0.961, with optimal cutoffs of 0.309 and 0.386. Compared with the conventionally used cutoff of 1, the optimal cutoffs substantially improved sensitivity while maintaining high specificity, particularly for IAS. Insulin concentration alone also showed good diagnostic performance for distinguishing IAS from insulinoma, with AUCs of 0.985 and 0.943 and optimal cutoffs of 83.1 and 58.86 μIU/mL during hypoglycemic episodes and in the fasting state, respectively, but was less informative for EIAS, with AUCs of 0.853 and 0.729 and optimal cutoffs of 67.85 and 71.94 μIU/mL, respectively.

**Conclusion:**

The insulin-to-C-peptide molar ratio, measured either during hypoglycemic episodes or in the fasting state, showed good diagnostic performance for distinguishing IAS and EIAS from insulinoma. The conventionally used cutoff of 1 appears to be too high, and lower cutoffs may provide better sensitivity while preserving specificity. Validation in larger cohorts is needed.

## 1 Introduction

Insulin autoimmune syndrome (IAS) is characterized by spontaneous hypoglycemia or marked glycemic fluctuations due to inappropriate binding and dissociation of insulin autoantibodies (IAA) with insulin in individuals without prior exposure to exogenous insulin (1, 2). Exogenous insulin antibody syndrome (EIAS) is a condition occurs in patients previously exposed to exogenous insulin and share similar clinical and biochemical features with those of IAS (3). Although IAS and EIAS have been reported more frequently in East Asian populations, cases have been described worldwide (4–6). Failure to recognize IAS and EIAS in a timely manner may lead to severe hypoglycemia and unnecessary investigations, including pancreatic imaging or even surgical exploration for suspected insulinoma, thereby increasing medical costs and exposing patients to avoidable procedural risks (7, 8).

The diagnosis of IAS depends on an integrated assessment of clinical and laboratory findings (2, 9). Detection of IAA is an important diagnostic tool, but false-negative and false-positive results may occur (10). Polyethylene glycol (PEG) precipitation can be used to indirectly demonstrate the presence of insulin-IAA complexes, but its specificity is limited (11). Gel filtration chromatography is technically complicated and not widely available in routine clinical practice (12). Additional approaches are therefore needed to facilitate the diagnosis of IAS.

The dissociation between insulin and C-peptide levels is a characteristic biochemical feature of IAS and an important diagnostic clue (1, 13). Compared with the diagnostic approaches described above, the insulin-to-C-peptide molar ratio is more readily available in routine clinical practice. In healthy individuals, because insulin and C-peptide are secreted in equimolar amounts but insulin is cleared more rapidly than C-peptide, the peripheral insulin-to-C-peptide molar ratio is typically around 0.15 (14). In IAS, binding of insulin to IAA slows insulin clearance, leading to disproportionately elevated insulin concentrations relative to C-peptide. However, the optimal diagnostic cutoff remains unclear. A molar ratio greater than 1 has often been used as a suggestive threshold for IAS because insulin and C-peptide are secreted in equimolar amounts (1). However, this rationale is incomplete, as insulin undergoes first-pass hepatic clearance and has a shorter half-life than C-peptide. Moreover, not all patients meet this threshold. In a cohort of 16 hospitalized patients with IAS (15), only 10 had a molar ratio greater than 1, suggesting that 1 may not be the optimal cutoff.

Positive insulin antibodies (IA) results are common in patients treated with exogenous insulin, and approximately 26% of IA-positive patients may develop EIAS (16, 17). Although uncommon, insulinoma has also been reported in patients treated with exogenous insulin (18, 19). Accordingly, distinguishing EIAS from insulinoma is even more challenging than distinguishing IAS from insulinoma because different exogenous insulin preparations exhibit variable cross-reactivity with insulin immunoassays, resulting in substantial assay-dependent differences in measured insulin concentrations (20). In addition, patients with severe β-cell dysfunction may have extremely low or undetectable C-peptide levels, further complicating interpretation of the insulin-to-C-peptide molar ratio (21).

To our knowledge, no previous study has systematically evaluated the optimal insulin-to-C-peptide molar ratio cutoff for distinguishing IAS and EIAS from insulinoma. We therefore aimed to evaluate the diagnostic performance of the insulin-to-C-peptide molar ratio separately for distinguishing IAS and EIAS from insulinoma, the most common cause of endogenous hyperinsulinemic hypoglycemia, and to identify the optimal diagnostic cutoff for each condition.

## 2 Methods

The study was approved by the Ethics Committee of Peking Union Medical College Hospital.

### 2.1 Patients

19 patients with IAS and 33 patients with EIAS discharged from Peking Union Medical College Hospital between 2013 and 2025 were included in the study. The diagnosis of IAS was based on the following criteria: fulfillment of Whipple’s triad; biochemical findings consistent with endogenous hyperinsulinemic hypoglycemia; detection of high-titer IAA, or confirmation by PEG precipitation or gel filtration chromatography; and no prior exposure to exogenous insulin. The diagnosis of EIAS was based on the following criteria: recurrent hypoglycemic episodes not explained by insulin dose in patients with diabetes receiving exogenous insulin; no improvement in hypoglycemia after insulin dose reduction or discontinuation; and detection of high-titer IAA, or confirmation by PEG precipitation or gel filtration chromatography. For patients with diagnostic uncertainty, final inclusion was determined after discussion with at least two experienced endocrinologists. Patients were excluded if no hypoglycemic episode occurred during hospitalization or if hypoglycemia was suspected to be caused by surreptitious use of exogenous insulin or insulin secretagogues. In the EIAS group, patients receiving insulin detemir or insulin degludec were excluded because these insulin analogs are predominantly bound to albumin in the circulation, which may result in higher measured insulin concentrations than those of other insulin analogs at the same dose (22, 23).

The control group consisted of 70 patients with insulinoma who were hospitalized at Peking Union Medical College Hospital between 2013 and 2025 and had pathologically confirmed insulinoma after surgery. These patients were randomly selected from the eligible insulinoma population using computer-generated random numbers.

Hypoglycemia was defined as a venous plasma glucose level below 3.0 mmol/L in patients with IAS and below 3.9 mmol/L in patients with EIAS and diabetes (24, 25).

### 2.2 Data collection

Demographic data (age, sex, date of admission, height, and body weight), hypoglycemic pattern (timing of episodes, and the lowest documented glucose level), biochemical data (plasma glucose, insulin, C-peptide, proinsulin, and urinary ketones) during hypoglycemic episodes, ancillary investigations (IAA, PEG precipitation, glycated hemoglobin (HbA1c), and pancreatic imaging findings), and treatment were extracted from the medical records. For patients with IAS, data on potential triggers and comorbidities were also collected. For patients with EIAS, additional data including diabetes type and duration, the presence of insulin allergy, and the type of exogenous insulin used were collected.

Plasma glucose was measured by the glucose oxidase assay. Insulin and C-peptide were measured by chemiluminescence immunoassays on the Siemens ADVIA Centaur XP system (Catalog # 02230141, RRID: AB_2909499; Catalog # 03649928, RRID: AB_2909501) before August 2021 and on the Siemens Atellica system thereafter (Catalog # 10995628, RRID: AB_2941780; Catalog # 10995541, RRID: AB_2941781). The clinical laboratory confirmed assay comparability between the two platforms. The assay measuring ranges were 1-300 μIU/mL for insulin and 0.05–30 ng/mL for C-peptide. If a result fell outside the detectable range and was not remeasured after dilution, the upper or lower limit of the range was used for calculation of the insulin-to-C-peptide molar ratio. For unit conversion, insulin was converted as 1 μIU/mL = 6.945 pmol/L, and C-peptide as 1 ng/mL = 331 pmol/L. Between 2013 and 2025, the methods used for IAA measurement at Peking Union Medical College Hospital changed over time, including enzyme linked immunosorbent assay and radioimmunoassay. HbA1c was measured by high-performance liquid chromatography. For PEG precipitation, insulin concentration was measured in the supernatant and compared with the total insulin concentration before precipitation.

Among the 19 patients with IAS, biochemical data during hypoglycemic episodes were available for all patients and biochemical data at fasting for 18 patients. Among the 33 patients with EIAS, biochemical data during hypoglycemic episodes were available for 18 patients and biochemical data at fasting for 30 patients. Among 70 patients with insulinoma, biochemical data during hypoglycemic episodes were available for 65 patients and biochemical data at fasting for 60 patients.

### 2.3 Statistical analysis

Continuous variables are presented as medians with interquartile ranges (IQRs), and categorical variables are presented as counts and percentages. All comparisons were performed separately between two groups, i.e. IAS versus insulinoma and EIAS versus insulinoma. Clinical characteristics were compared using the Mann-Whitney U test for continuous variables and the chi-square test for categorical variables. In the primary analysis, receiver-operating-characteristic (ROC) curve analysis was performed to evaluate the diagnostic performance of the insulin-to-C-peptide molar ratio for distinguishing IAS from insulinoma and EIAS from insulinoma. The area under the ROC curve (AUC) was calculated, and the optimal cutoff value was determined using the Youden index.

Additional ROC analyses were performed to evaluate the diagnostic performance of insulin concentration alone for distinguishing IAS from insulinoma and EIAS from insulinoma. To further reduce imbalance in insulin concentrations between groups, a *post hoc* ROC analysis was performed using patients with pathologically confirmed insulinoma who had relatively high insulin concentrations, defined as insulin concentrations greater than 50 μIU/mL during hypoglycemic episodes, as controls. Sensitivity analyses were also conducted by excluding: (1) patients with EIAS who were still receiving exogenous insulin during hospitalization; (2) samples obtained during postprandial hypoglycemia; and (3) patients without definitively positive PEG precipitation results, defined as a decrease of more than 40% in insulin concentration after PEG precipitation compared with the total insulin concentration before precipitation (2). Spearman correlation analysis was used to assess the association between the insulin-to-C-peptide molar ratio and body mass index (BMI) in all three groups. All statistical analyses were performed using R software (version 4.3.3). A two-sided P value of less than 0.05 was considered statistically significant. Missing data were not imputed, and each analysis was performed using the available data for the corresponding time point.

## 3 Results

### 3.1 Study population and clinical characteristics

19 patients with IAS, 33 with EIAS, and 70 with pathologically confirmed insulinoma were included. Clinical characteristics are summarized in **Table 1** and **Supplementary Table 1**. Compared with insulinoma, IAS was associated with older age, higher HbA1c, and higher fasting plasma glucose. Insulin, C-peptide, and insulin-to-C-peptide molar ratios were higher in IAS than in insulinoma in both the fasting state and during hypoglycemic episodes. Compared with insulinoma, EIAS was associated with older age, lower BMI, higher HbA1c, and higher plasma glucose. In both the fasting state and during hypoglycemic episodes, EIAS was associated with higher insulin concentrations and insulin-to-C-peptide molar ratios, but lower C-peptide concentrations.

**Table 1:** Clinical characteristics of patients with IAS, EIAS, and insulinoma.

Characteristic	IAS (n=19)	EIAS (n=33)	Insulinoma (n=70)
Age (years)	55.00 (52.50-65.00)	58.00 (47.00-62.00)	49.00 (35.50-57.00)
Female (n, %)	10 (52.6%)	19 (57.6%)	43 (61.4%)
BMI (kg/m^2^)	26.56 (25.07-30.06)	22.68 (20.26-23.83)	27.86 (24.45-31.20)
HbA1c (%)	5.70 (5.15-6.10)	8.20 (7.10-9.20)	4.70 (4.43-5.00)
Fasting plasma glucose (mmol/L)	3.95 (2.80-4.60)	6.50 (4.53-10.03)	2.70 (2.12-3.55)
Fasting insulin (μIU/mL)	155.32 (97.12-296.38)	62.49 (27.35-167.30)	19.40 (13.65-44.95)
Fasting C-peptide (ng/mL)	4.57 (3.83-7.84)	0.41 (0.06-1.99)	2.72 (1.70-4.33)
Fasting insulin/C-peptide molar ratio	0.72 (0.50-1.45)	3.69 (0.87-10.09)	0.17 (0.14-0.24)
During hypoglycemic episode			
Plasma glucose (mmol/L)	2.30 (1.90-2.70)	2.65 (2.50-2.90)	2.30 (1.90-2.60)
Insulin (μIU/mL)	275.48 (172.85-900.00)	137.00 (45.01-209.12)	23.23 (13.40-49.70)
C-peptide (ng/mL)	6.01 (4.34-8.84)	0.29 (0.05-2.87)	3.06 (1.96-4.67)
Insulin/C-peptide molar ratio	0.93 (0.64-3.24)	10.77 (1.58-21.03)	0.17 (0.14-0.23)

Data are presented as median (interquartile range) or n (%).

For IAS versus insulinoma, no significant between-group differences were observed in the proportion of women, BMI, fasting proinsulin, or plasma glucose and proinsulin during hypoglycemic episodes (P>0.05); the remaining variables differed significantly (P<0.05). For EIAS versus insulinoma, the proportion of women did not differ significantly (P>0.05), whereas the remaining variables differed significantly (P<0.05).

IAS, insulin autoimmune syndrome; EIAS, exogenous insulin antibody syndrome; BMI, body mass index; HbA1c, glycated hemoglobin.

Additional clinical features, including potential triggers, comorbidities, hypoglycemic patterns, and ancillary investigations, are summarized in **Supplementary Tables 2** and **3**. Among patients with IAS, 17 of 19 (89.5%) were IAA-positive. Eight patients had PEG precipitation results, with a median decrease in insulin concentration after PEG precipitation of 93.3% (IQR, 75.4%-96.0%). Among patients with EIAS, 30 of 33 (90.9%) were IAA-positive. Six patients had PEG precipitation results, with a median decrease in insulin concentration after PEG precipitation of 83.4% (IQR, 42.5%-93.3%). Among 70 patients with insulinoma, 37 had available IAA results, of whom 94.6% (35/37) were negative.

To reduce imbalance in insulin concentrations between groups, we further compared IAS and EIAS with 29 patients with pathologically confirmed insulinoma who had relatively high insulin concentrations. Of these, 16 were from the randomly selected 70 insulinoma controls, and 13 were additional cases outside the random sample. All cases were from Peking Union Medical College Hospital. Clinical characteristics are summarized in **Supplementary Table 4**. Between-group differences in insulin concentrations were reduced but not eliminated in the IAS versus insulinoma comparison. Insulin concentrations in both the fasting state and during hypoglycemic episodes no longer differed significantly between EIAS and insulinoma. Notably, this high-insulin insulinoma group had higher BMI and higher insulin-to-C-peptide molar ratios than the overall randomly selected insulinoma group.

### 3.2 Diagnostic performance of the insulin-to-C-peptide molar ratio

**Figure 1:**
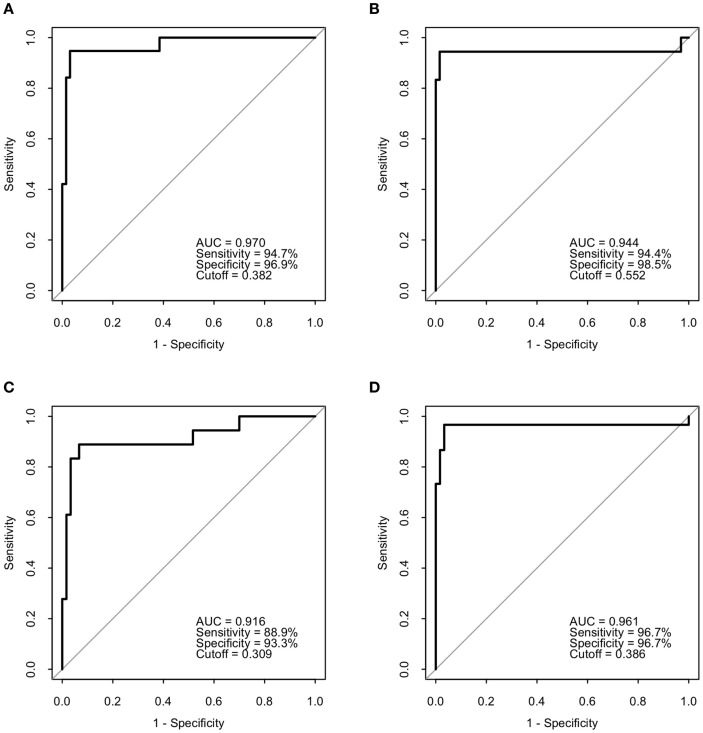
ROC curves of the insulin-to-C-peptide molar ratio for distinguishing IAS and EIAS from insulinoma. ROC curves are shown for IAS versus insulinoma during hypoglycemic episodes **(A)** and in the fasting state **(C)**, and for EIAS versus insulinoma during hypoglycemic episodes **(B)** and in the fasting state **(D)**. ROC, receiver-operating-characteristic; IAS, insulin autoimmune syndrome; EIAS, exogenous insulin antibody syndrome; AUC, area under the receiver-operating-characteristic curve.

ROC analysis showed good diagnostic performance of the insulin-to-C-peptide molar ratio for distinguishing IAS and EIAS from insulinoma at both time points (**Figure 1**; **Table 2**). During hypoglycemic episodes, the AUC was 0.970 for IAS versus insulinoma, with an optimal cutoff of 0.382, sensitivity of 94.7%, and specificity of 96.9% (**Figure 1A**); the corresponding values for EIAS versus insulinoma were 0.944, 0.552, 94.4%, and 98.5%, respectively (**Figure 1B**). In the fasting state, the AUC was 0.916 for IAS versus insulinoma, with an optimal cutoff of 0.309, sensitivity of 88.9%, and specificity of 93.3% (**Figure 1C**); the corresponding values for EIAS versus insulinoma were 0.961, 0.386, 96.7%, and 96.7%, respectively (**Figure 1D**). The optimal cutoffs were consistently lower than the conventionally used cutoff of 1 and yielded markedly higher sensitivity while maintaining high specificity (**Table 2**).

**Table 2:** Comparison of sensitivity and specificity at the optimal cutoff and at the conventionally used cutoff of 1 for the insulin-to-C-peptide molar ratio in distinguishing IAS and EIAS from insulinoma.

Comparison	Time point	AUC	Optimal cutoff			Conventional cutoff of 1		Sample size
			Cutoff value	Sensitivity	Specificity	Sensitivity	Specificity	
IAS vs Insulinoma	During hypoglycemic episode	0.970	0.382	0.947	0.969	0.421	0.985	84
	Fasting	0.916	0.309	0.889	0.933	0.333	0.983	78
EIAS vs Insulinoma	During hypoglycemic episode	0.944	0.552	0.944	0.985	0.833	0.985	83
	Fasting	0.961	0.386	0.967	0.967	0.733	0.983	90

The optimal cutoff was determined using the Youden index.

IAS, insulin autoimmune syndrome; EIAS, exogenous insulin antibody syndrome; AUC, area under the receiver-operating-characteristic curve.

**Figure 2:**
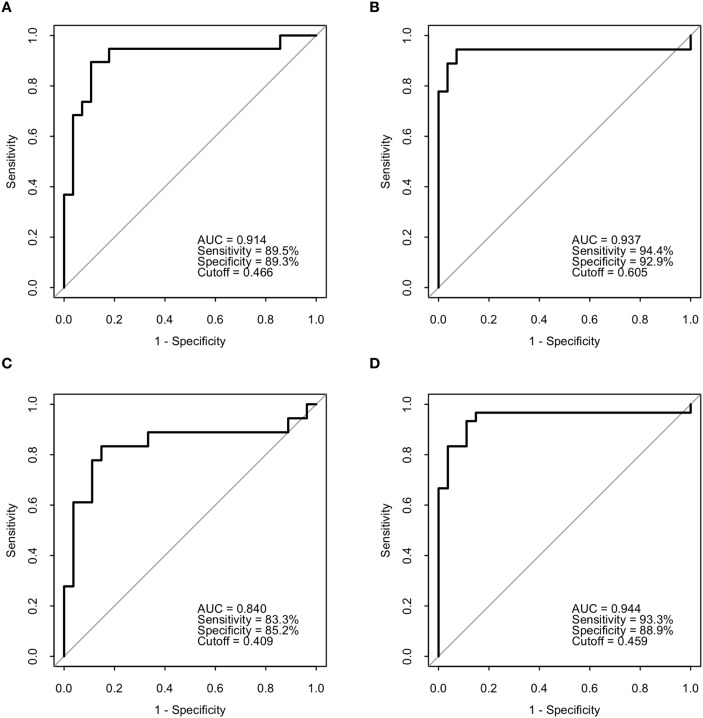
ROC curves of the insulin-to-C-peptide molar ratio for distinguishing IAS and EIAS from insulinoma with relatively high insulin concentrations. ROC curves are shown for IAS versus insulinoma during hypoglycemic episodes **(A)** and in the fasting state **(C)**, and for EIAS versus insulinoma during hypoglycemic episodes **(B)** and in the fasting state **(D)**. Relatively high insulin concentrations were defined as insulin concentrations greater than 50 μIU/mL during hypoglycemic episodes. ROC, receiver-operating-characteristic; IAS, insulin autoimmune syndrome; EIAS, exogenous insulin antibody syndrome; AUC, area under the receiver-operating-characteristic curve.

When compared with insulinoma cases with relatively high insulin concentrations, the insulin-to-C-peptide molar ratio remained discriminatory, with AUCs of 0.914 and 0.840 for IAS versus insulinoma during hypoglycemic episodes and in the fasting state, respectively, and 0.937 and 0.944 for EIAS versus insulinoma (**Figure 2**). The optimal cutoffs were similar to those in the primary analysis.

Sensitivity analyses yielded AUCs and optimal cutoffs similar to those of the primary analysis (**Supplementary Table 5**). The only exception was the analysis excluding patients without definitively positive PEG precipitation results, in which the optimal cutoff for EIAS versus insulinoma increased to 1.434 during hypoglycemic episodes and 1.625 in the fasting state; however, these estimates were based on small sample sizes, with only 3 and 4 EIAS samples, respectively.

### 3.3 Diagnostic performance of insulin concentration

**Figure 3:**
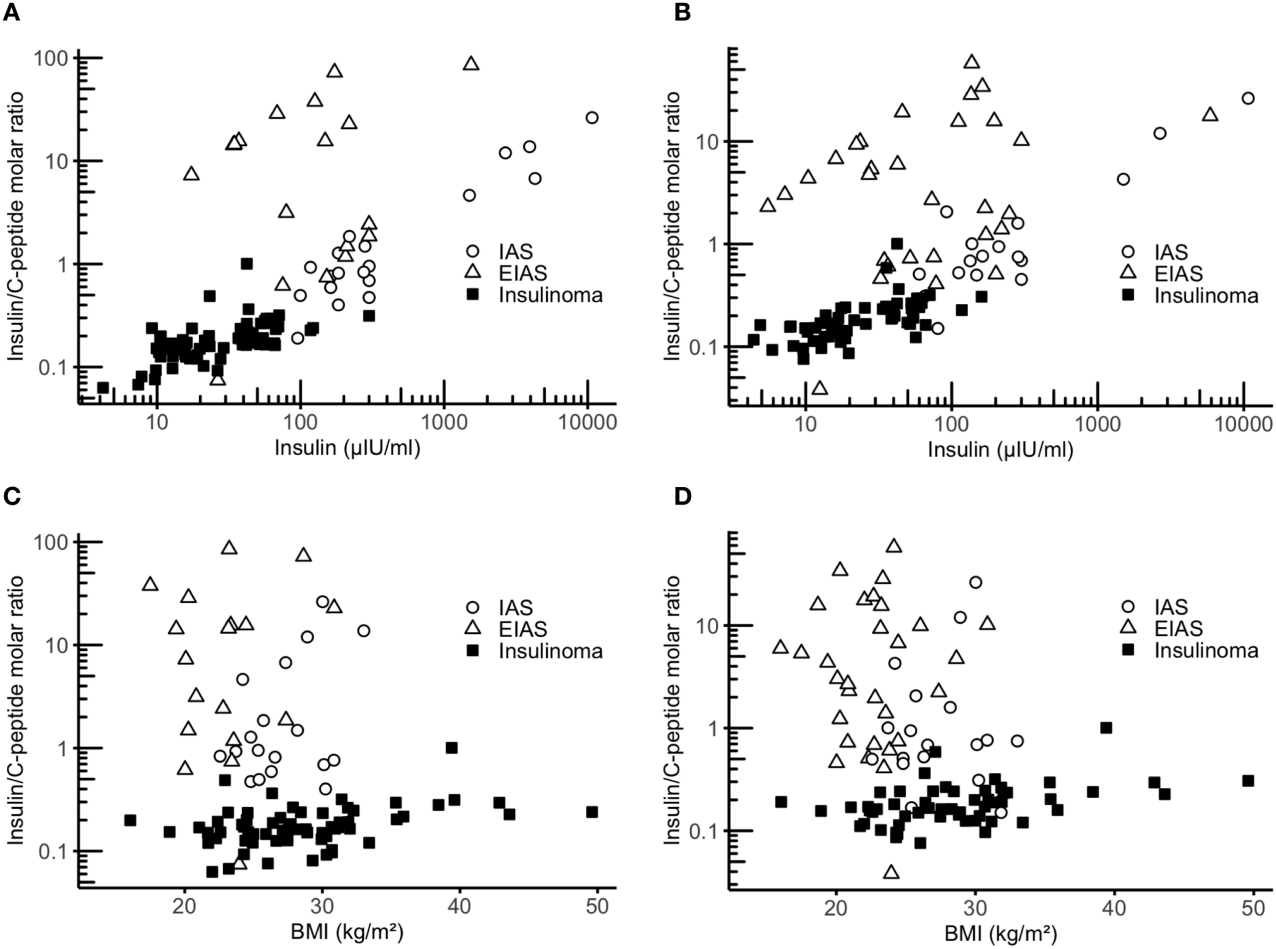
Scatter plots illustrating the relationship between the insulin-to-C-peptide molar ratio and insulin concentration during hypoglycemic episodes **(A)** and in the fasting state **(B)**, and the relationship between the insulin-to-C-peptide molar ratio and BMI during hypoglycemic episodes **(C)** and in the fasting state **(D)**. Data are shown for patients with IAS, EIAS, and insulinoma. BMI, body mass index; IAS, insulin autoimmune syndrome; EIAS, exogenous insulin antibody syndrome.

**Figure 4:**
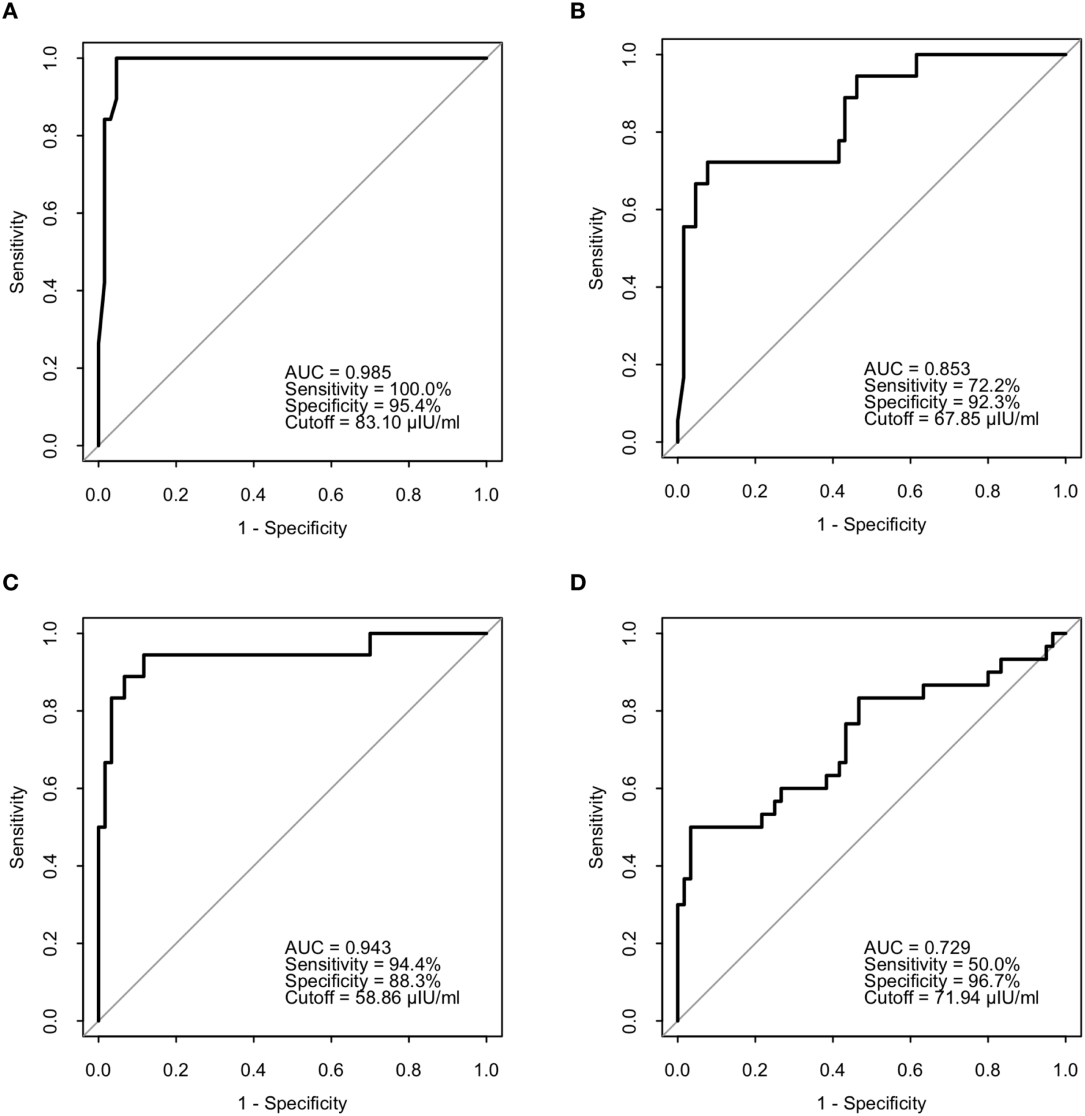
ROC curves of insulin concentration for distinguishing IAS and EIAS from insulinoma. ROC curves are shown for IAS versus insulinoma during hypoglycemic episodes **(A)** and in the fasting state **(C)**, and for EIAS versus insulinoma during hypoglycemic episodes **(B)** and in the fasting state **(D)**. ROC, receiver-operating-characteristic; IAS, insulin autoimmune syndrome; EIAS, exogenous insulin antibody syndrome; AUC, area under the receiver-operating-characteristic curve.

In scatter plots of the insulin-to-C-peptide molar ratio and insulin concentration (**Figures 3A, B**), IAS and insulinoma were separated not only by the molar ratio but also by insulin concentration itself, whereas EIAS and insulinoma showed greater overlap in insulin concentration. We therefore further evaluated the diagnostic performance of insulin concentration alone. During hypoglycemic episodes, the AUC was 0.985 for IAS versus insulinoma, with an optimal cutoff of 83.1 μIU/mL (**Figure 4A**), and 0.853 for EIAS versus insulinoma, with an optimal cutoff of 67.85 μIU/mL (**Figure 4B**). In the fasting state, the corresponding AUCs were 0.943 and 0.729, with optimal cutoffs of 58.86 and 71.94 μIU/mL, respectively (**Figures 4C, D**).

### 3.4 Correlation between the molar ratio and BMI

Scatter plots of the insulin-to-C-peptide molar ratio and BMI showed separation of IAS and EIAS from insulinoma regardless of BMI (**Figures 3C, D**). In insulinoma, BMI appeared to show a linear association with the insulin-to-C-peptide molar ratio, whereas no clear relationship was observed in IAS or EIAS. Spearman analysis confirmed a modest positive correlation in insulinoma during hypoglycemic episodes (rho=0.3687, P = 0.0029) and in the fasting state (rho=0.4324, P = 0.0009), but no significant correlation in IAS or EIAS at either time point (**Supplementary Table 6**).

## 4 Discussion

In this retrospective study, the insulin-to-C-peptide molar ratio, measured either during hypoglycemic episodes or in the fasting state, showed good diagnostic performance for distinguishing IAS and EIAS from insulinoma. Across the primary and sensitivity analyses, the optimal cutoffs were consistently lower than the conventionally used cutoff of 1. Although a cutoff of 1 preserved high specificity, its sensitivity was suboptimal. Lower cutoffs substantially improved sensitivity while maintaining high specificity, particularly for IAS. In addition, insulin concentration alone showed good discriminatory performance for IAS versus insulinoma, suggesting that high insulin concentration may also provide useful diagnostic information in selected clinical settings.

IAS and insulinoma share the biochemical phenotype of hyperinsulinemic hypoglycemia and can be difficult to distinguish in some cases, but their management differs fundamentally. Suspected insulinoma often leads to pancreatic imaging and may ultimately require surgery, whereas IAS is often self-limited, is mainly managed with supportive measures, and in selected cases requires immunosuppressive therapy. Delayed recognition of IAS may therefore expose patients to unnecessary and costly investigations or invasive procedures. Our findings support the insulin-to-C-peptide molar ratio as a useful adjunct in the differential diagnosis of hyperinsulinemic hypoglycemia.

Although most previously reported IAS cases have shown a molar ratio above the conventionally used cutoff of 1 (1, 13, 26), ratios below 1 have also been reported (15, 27, 28). Our results indicate that the conventionally used cutoff of 1 is too high for a substantial proportion of patients. Accordingly, a molar ratio below 1 should not be used to exclude IAS in clinical practice. Several factors may contribute to this finding, including antibody binding to C-peptide or proinsulin, interference of insulin-IAA complexes on insulin and C-peptide measurement, inter-assay variability, and a less marked degree of insulin-C-peptide dissociation in some patients, potentially reflecting a milder biochemical phenotype (9, 29, 30). In our cohort, the optimal cutoff for distinguishing IAS from insulinoma ranged from 0.30 to 0.65, although external validation will be required before this range can be generalized.

The insulin-to-C-peptide molar ratio during hypoglycemic episodes is generally considered diagnostically informative. Our results suggest that the fasting molar ratio may also provide useful diagnostic information when hypoglycemic samples are unavailable. This finding is plausible because, even in the absence of hypoglycemia, insulin may remain bound to insulin autoantibodies, leading to disproportionately elevated measured insulin relative to C-peptide.

We also found that insulin concentration alone had important diagnostic value for distinguishing IAS from insulinoma. However, because PEG precipitation and gel filtration chromatography were not routinely available, the diagnosis of IAS at our center was made particularly cautiously in patients without marked hyperinsulinemia. As a result, our cohort may underrepresent IAS cases with relatively lower insulin concentrations, potentially leading to overestimation of the diagnostic value of insulin concentration.

EIAS shares many clinical and biochemical features with IAS, but the diagnosis is even more challenging because measured insulin may be influenced by exogenous insulin preparations as well as by varying degrees of β-cell dysfunction. In our study, as in IAS, the insulin-to-C-peptide molar ratio in EIAS showed good diagnostic performance both during hypoglycemic episodes and in the fasting state, and the optimal cutoffs were also below 1, approximately 0.35 to 0.60. A higher cutoff was observed in the sensitivity analysis restricted to patients with definitively positive PEG precipitation results, but this estimate was based on very few EIAS samples and should be interpreted cautiously. By contrast, insulin concentration alone performed less satisfactorily for distinguishing EIAS from insulinoma, possibly because measured insulin is more strongly affected by the type of exogenous insulin preparation and assay cross-reactivity.

An additional finding was the positive correlation between BMI and the insulin-to-C-peptide molar ratio in insulinoma. Consistent with this observation, the high-insulin insulinoma group also had higher BMI and higher molar ratios than the overall randomly selected insulinoma group. Notably, 2 patients with insulinoma had a molar ratio greater than 1, and both had relatively high BMI values (39.4 and 34.2 kg/m^2^), indicating that the molar ratio may be elevated in insulinoma patients with higher BMI. This pattern is consistent with previous studies and suggests that excessive insulin promote adipogenesis, whereas obesity may in turn increase the molar ratio by reducing hepatic insulin clearance (31, 32). By contrast, no association between BMI and the molar ratio was observed in IAS or EIAS. A possible explanation is that the adipogenic effect of insulin may be disrupted by binding to IAA or IA, and the elevated molar ratio is mainly driven by binding of IAA or IA to insulin rather than by obesity-related insulin resistance.

We also observed that insulinoma was associated with lower fasting plasma glucose and HbA1c levels than IAS and EIAS, despite hypoglycemia being present in all groups. This pattern suggests that patients with IAS or EIAS may experience greater glycemic fluctuations, and continuous glucose monitoring may provide additional information on glycemic variability and aid in the differential diagnosis.

This study has several limitations. First, it was a single-center study with a relatively small sample size, especially for IAS, which may limit the generalizability of the findings. Second, all insulin and C-peptide measurements were obtained using a single assay platform. Given the known variability in assay performance (33), the diagnostic cutoffs identified here may not be directly applicable to other laboratories. Although the assays used at our center are among the mainstream commercially available immunoassays, accurate insulin measurement is still challenging because IAA or IA may interfere with standard immunoassays, and in EIAS the contribution of exogenous insulin further complicates interpretation. As the association and dissociation of insulin with IAA or IA may vary under different physiological conditions, measured insulin concentrations may also fluctuate over time. Third, clinical characteristics differed across groups, including age, BMI, and HbA1c, and these differences may have confounded the results. Fourth, because PEG precipitation was not performed in all patients, corresponding results were unavailable for a proportion of cases, and long-term follow-up data were limited. These factors may have affected diagnostic certainty in some cases. However, all control patients had pathologically confirmed insulinoma, providing a definitive reference diagnosis. Finally, although we performed *post hoc* analysis using insulinoma cases with relatively high insulin concentrations, we failed to evaluate the diagnostic performance of the molar ratio in IAS and insulinoma patients with fully comparable insulin concentrations.

## 5 Conclusion

In conclusion, the insulin-to-C-peptide molar ratio, measured either during hypoglycemic episodes or in the fasting state, showed good diagnostic performance for distinguishing IAS and EIAS from insulinoma. A cutoff of 1 provided high specificity but insufficient sensitivity, lower cutoffs improved sensitivity while preserving specificity, particularly for IAS. Insulin concentration alone may also be informative for differentiating IAS from insulinoma. These findings support the diagnostic value of the insulin-to-C-peptide molar ratio in the differential diagnosis of hyperinsulinemic hypoglycemia, particularly when specialized diagnostic tests are unavailable, and indicate that the conventionally used cutoff of 1 may not be optimal, although validation in larger cohorts is needed.

## Data Availability

The raw data supporting the conclusions of this article will be made available by the authors, without undue reservation.
