# Supplementary material for: Diagnostic utility of the insulin-to-C-peptide molar ratio in differentiating insulin autoimmune syndrome and exogenous insulin antibody syndrome from insulinoma
Source: Front Endocrinol (Lausanne). 2026 Jul 7;17:1879250. doi: 10.3389/fendo.2026.1879250 (PMC13385051; doi:10.3389/fendo.2026.1879250)
Supplement: Supplementary file 1 [file Table1.pdf]

Supplementary Material

Supplementary Table 1. P values for comparisons of clinical characteristics between IAS and insulinoma and between EIAS and insulinoma

Supplementary Table 2. Additional clinical characteristics of patients with IAS

Supplementary Table 3. Additional clinical characteristics of patients with EIAS

Supplementary Table 4. Clinical characteristics of patients with IAS, EIAS, and insulinoma with relatively high insulin concentrations

Supplementary Table 5. Sensitivity analyses of the diagnostic performance of the insulin-to-C-peptide molar ratio for distinguishing IAS and EIAS from insulinoma

Supplementary Table 6. Spearman correlations between the insulin-to-C-peptide molar ratio and body mass index

Supplementary Table 1. P values for comparisons of clinical characteristics between IAS and insulinoma and between EIAS and insulinoma

Characteristic	P value (IAS vs Insulinoma)	P value (EIAS vs Insulinoma)
Age (years)	0.012	0.029
Female (n, %)	0.668	0.875
BMI (kg/m^2)	0.553	<0.001
HbA1c (%)	<0.001	<0.001
Fasting plasma glucose (mmol/L)	0.030	<0.001
Fasting insulin (μIU/mL)	<0.001	<0.001
Fasting C-peptide (ng/mL)	0.002	<0.001
Fasting insulin/C-peptide molar ratio	<0.001	<0.001
**During hypoglycemic episode**		
Plasma glucose (mmol/L)	0.944	0.002
Insulin (μIU/mL)	<0.001	<0.001
C-peptide (ng/mL)	<0.001	<0.001
Insulin/C-peptide molar ratio	<0.001	<0.001

P values were calculated using the Mann–Whitney U test for continuous variables and the chi-square test for categorical variables.

Abbreviations: IAS, insulin autoimmune syndrome; EIAS, exogenous insulin antibody syndrome; BMI, body mass index; HbA1c, glycated hemoglobin.

Supplementary Table 2. Additional clinical characteristics of patients with IAS

Characteristic	IAS (n=19)
**Diabetes (n, %)**	1 (5.3%)
Type 2 diabetes	1 (5.3%)
Diabetes duration (years)	9
**Autoimmune or hematological disorders (n, %)**	9 (47.4%)
Hashimoto's thyroiditis	1 (5.3%)
Graves disease	3 (15.8%)
Systemic lupus erythematosus	1 (5.3%)
Rheumatoid arthritis	2 (10.5%)
Autoimmune hemolytic anemia	1 (5.3%)
Monoclonal gammopathy of undetermined significance	1 (5.3%)
**Sulfhydryl-containing drug exposure (n, %)**	10 (52.6%)*
Antithyroid drugs	3 (15.8%)
Methimazole	3 (15.8%)
Supplements	2 (10.5%)
Alpha-lipoic acid	1 (5.3%)
Glutathione	1 (5.3%)
Antiplatelet drugs	3 (15.8%)
Clopidogrel	3 (15.8%)
Proton pump inhibitors	3 (15.8%)
Pantoprazole	1 (5.3%)
Omeprazole	2 (10.5%)
Antibiotics	1 (5.3%)
Penicillin	1 (5.3%)
**Viral infection (n, %)**	3 (15.8%)
varicella zoster virus	1 (5.3%)
COVID-19	2 (10.5%)
Allergic history (n, %)	5 (26.3%)
**Hypoglycemic pattern (n, %)**	
Fasting only	14 (73.7%)
Both fasting and postprandial	5 (26.3%)
Postprandial only	0 (0%)
Alternation between hyperglycemia and hypoglycemia (n, %)	13 (68.4%)
Lowest plasma glucose (mmol/L)	1.82 (1.40-2.29)
Insulin change after polyethylene glycol precipitation (n=8)	93.3% (75.4%-96.0%)
Insulin autoantibody positivity (n, %)	17 (89.5%)
Negative pancreatic imaging** (n=17)	17 (100.0%)
Treatment (n, %)	
Dietary intervention	19 (100%)
Sulfhydryl-containing drug discontinuation (n=10)	10 (100%)
Alpha-glucosidase inhibitor	12 (63.2%)
Glucocorticoid	6 (31.6%)

Immunosuppressive agents	2 (10.5%)[#]
Others[##]	1 (5.3%)

Data are presented as median (interquartile range) or n (%).

[*] Two patients were exposed to two sulfhydryl-containing drugs.

[**] Pancreatic imaging included contrast-enhanced pancreatic or abdominal computed tomography or magnetic resonance imaging, somatostatin receptor imaging, or glucagon-like peptide-1 receptor imaging, with at least one of them showing negative results.

[#] Two patients receiving immunosuppressive agents had comorbid systemic lupus erythematosus or rheumatoid arthritis.

[##] One patient with concomitant monoclonal gammopathy of undetermined significance was treated with ixazomib, lenalidomide, and dexamethasone.

Abbreviations: IAS, insulin autoimmune syndrome.

Supplementary Table 3. Additional clinical characteristics of patients with EIAS

Characteristic	EIAS (n=33)
**Diabetes (n, %)**	33 (100%)
Type 2 diabetes	19 (57.6%)
Classic type 1 diabetes	9 (27.3%)
Fulminant type 1 diabetes	2 (6.1%)
Latent autoimmune diabetes of adults	3 (9.1%)
Diabetes duration (years)	8.4 (4.7-16.5)
**Autoimmune or hematological disorders (n, %)**	10 (30.3%)*
Hashimoto's thyroiditis	5 (15.2%)
Graves disease	1 (3.0%)
IgG4-related disease	3 (9.1%)
Primary biliary cholangitis	1 (3.0%)
Drug-induced hypersensitivity syndrome	2 (6.1%)
Allergic history (n, %)	17 (51.5%)
Insulin Allergy (n, %)	8 (24.2%)
**Insulin formulation (n, %)**	
Human insulin	8 (24.2%)
Insulin analog	17 (51.5%)
Human insulin + insulin analog	8 (24.2%)
**Hypoglycemic pattern (n, %)**	
Fasting only	22 (66.7%)
Both fasting and postprandial	11 (33.3%)
Postprandial only	0 (0%)
Alternation between hyperglycemia and hypoglycemia (n, %)	30 (90.9%)
Lowest plasma glucose (mmol/L)	2.3 (2.0-2.9)
Insulin change after polyethylene glycol precipitation (n=6)	83.4% (42.5%-93.3%)
Insulin autoantibody positivity (n, %)	30 (90.9%)
Negative pancreatic imaging** (n=14)	14 (100%)
**Treatment (n, %)**	
Dietary intervention	33 (100%)
Insulin discontinuation	10 (30.3%)
Insulin formulation switch	14 (42.4%)
Alpha-glucosidase inhibitor	16 (48.5%)
Glucocorticoid	8 (24.2%)
Immune suppressive drug	5 (15.2%)
Plasmapheresis	1 (3.0%)
Others#	1 (3.0%)

Data are presented as median (interquartile range) or n (%).

* Two patients had concomitant Hashimoto's thyroiditis and drug-induced hypersensitivity syndrome.

** Pancreatic imaging included contrast-enhanced pancreatic or abdominal computed tomography or

magnetic resonance imaging, somatostatin receptor imaging, or glucagon-like peptide-1 receptor imaging, with at least one of them showing negative results.

One patient with concomitant IgG4-related disease was treated with rituximab and baricitinib.

Abbreviations: EIAS, exogenous insulin antibody syndrome.

Supplementary Table 4. Clinical characteristics of patients with IAS, EIAS, and insulinoma with relatively high insulin concentrations

Characteristic	IAS (n=19)	EIAS (n=33)	Insulinoma (n=29)	P value (IAS vs Insulinoma)	P value (EIAS vs Insulinoma)
Age (years)	55.00 (52.50-65.00)	58.00 (47.00-62.00)	50.00 (42.00-55.38)	0.033	0.051
Female (n, %)	10 (52.6%)	19 (57.6%)	16 (55.2%)	1.000	1.000
BMI (kg/m^2)	26.56 (25.07-30.06)	22.68 (20.26-23.83)	31.96 (30.19-37.24)	<0.001	<0.001
HbA1c (%)	5.70 (5.15-6.10)	8.20 (7.10-9.20)	4.50 (4.25-5.05)	<0.001	<0.001
Fasting plasma glucose (mmol/L)	3.95 (2.80-4.60)	6.50 (4.53-10.03)	2.20 (1.95-2.85)	0.002	<0.001
Fasting insulin (μIU/mL)	155.32 (97.12-296.38)	62.49 (27.35-167.30)	60.93 (53.89-74.06)	<0.001	0.584
Fasting C-peptide (ng/mL)	4.57 (3.83-7.84)	0.41 (0.06-1.99)	5.19 (4.15-6.72)	0.763	<0.001
Fasting insulin/C-peptide molar ratio	0.72 (0.50-1.45)	3.69 (0.87-10.09)	0.27 (0.23-0.34)	<0.001	<0.001
**During hypoglycemic episode**					
Plasma glucose (mmol/L)	2.30 (1.90-2.70)	2.65 (2.50-2.90)	2.05 (1.80-2.40)	0.177	<0.001
Insulin (μIU/mL)	275.48 (172.85-900.00)	137.00 (45.01-209.12)	65.84 (57.79-79.78)	<0.001	0.123
C-peptide (ng/mL)	6.01 (4.34-8.84)	0.29 (0.05-2.87)	5.94 (4.34-6.71)	0.672	<0.001
Insulin/C-peptide molar ratio	0.93 (0.64-3.24)	10.77 (1.58-21.03)	0.26 (0.22-0.32)	<0.001	<0.001

Data are presented as median (interquartile range) or n (%).

Relatively high insulin concentrations were defined as insulin concentrations greater than 50 μIU/mL during hypoglycemic episodes.

P values were calculated for pairwise comparisons between IAS and insulinoma and between EIAS and insulinoma using the Mann–Whitney U test for continuous variables and the chi-square test for categorical variables.

Abbreviations: IAS, insulin autoimmune syndrome; EIAS, exogenous insulin antibody syndrome; BMI, body mass index; HbA1c, glycated hemoglobin.

Supplementary Table 5. Sensitivity analyses of the diagnostic performance of the insulin-to-C-peptide molar ratio for distinguishing IAS and EIAS from insulinoma

Sensitivity analysis	Comparison	Time point	AUC	Optimal cutoff	Sensitivity	Specificity	Sample size
Sensitivity analysis 1	EIAS vs Insulinoma	During hypoglycemic episode	0.875	0.552	0.875	0.985	73
		Fasting	0.906	0.386	0.917	0.967	72
Sensitivity analysis 2	IAS vs Insulinoma	During hypoglycemic episode	0.947	0.419	0.900	0.980	61
	EIAS vs Insulinoma	During hypoglycemic episode	0.900	0.553	0.900	0.980	61
Sensitivity analysis 3	IAS vs Insulinoma	During hypoglycemic episode	0.994	0.652	1.000	0.985	73
		Fasting	0.988	0.431	1.000	0.967	67
	EIAS vs Insulinoma	During hypoglycemic episode	1.000	1.434	1.000	1.000	68
		Fasting	1.000	1.625	1.000	1.000	64

Sensitivity analysis 1: excluding patients with EIAS who were still receiving exogenous insulin during hospitalization.

Sensitivity analysis 2: excluding samples obtained during postprandial hypoglycemia.

Sensitivity analysis 3: excluding patients without definitively positive PEG precipitation results.

Abbreviations: IAS, insulin autoimmune syndrome; EIAS, exogenous insulin antibody syndrome; AUC, area under the receiver-operating-characteristic curve.

Supplementary Table 6. Spearman correlations between the insulin-to-C-peptide molar ratio and body mass index

Group	Time point	rho	P value	Sample size
IAS	During hypoglycemic episode	-0.0132	0.9573	19
	Fasting	-0.0320	0.8997	18
EIAS	During hypoglycemic episode	0.0526	0.8370	18
	Fasting	-0.0011	0.9963	30
Insulinoma	During hypoglycemic episode	0.3687	0.0029	63
	Fasting	0.4324	0.0009	56

Abbreviations: IAS, insulin autoimmune syndrome; EIAS, exogenous insulin antibody syndrome.
